# Spatial–temporal distribution of incidence, mortality, and case-fatality ratios of coronavirus disease 2019 and its social determinants in Brazilian municipalities

**DOI:** 10.1038/s41598-023-31046-4

**Published:** 2023-03-13

**Authors:** Carlos Eduardo Raymundo, Marcella Cini Oliveira, Tatiana de Araujo Eleuterio, Édnei César de Arruda Santos Junior, Marcele Gonçalves da Silva, Suzana Rosa André, Ana Inês Sousa, Roberto de Andrade Medronho

**Affiliations:** 1 Instituto de Estudos em Saúde Coletiva, Universidade Federal do Rio de Janeiro, Avenida Horácio Macedo, 100 – Cidade Universitária, Rio de Janeiro, RJ CEP 21941-598 Brazil; 2 Faculdade de Medicina, Universidade Federal do Rio de Janeiro, Rio de Janeiro, RJ Brazil; 3 Faculdade de Enfermagem, Universidade Estadual do Rio de Janeiro, Rio de Janeiro, RJ Brazil; 4 Escola de Enfermagem Anna Nery, Universidade Federal do Rio de Janeiro, Rio de Janeiro, RJ Brazil

**Keywords:** Viral infection, Epidemiology

## Abstract

The COVID-19 pandemic caused impact on public health worldwide. Brazil gained prominence during the pandemic due to the magnitude of disease. This study aimed to evaluate the spatial–temporal dynamics of incidence, mortality, and case fatality of COVID-19 and its associations with social determinants in Brazilian municipalities and epidemiological week. We modeled incidence, mortality, and case fatality rates using spatial–temporal Bayesian model. “Bolsa Família Programme” (BOLSAFAM) and “proportional mortality ratio” (PMR) were inversely associated with the standardized incidence ratio (SIR), while “health insurance coverage” (HEALTHINSUR) and “Gini index” were directly associated with the SIR. BOLSAFAM and PMR were inversely associated with the standardized mortality ratio (SMR) and standardized case fatality ratio (SCFR). The highest proportion of excess risk for SIR and the SMR started in the North, expanding to the Midwest, Southeast, and South regions. The highest proportion of excess risk for the SCFR outcome was observed in some municipalities in the North region and in the other Brazilian regions. The COVID-19 incidence and mortality in municipalities that most benefited from the cash transfer programme and with better social development decreased. The municipalities with a higher proportion of non-whites had a higher risk of becoming ill and dying from the disease.

## Introduction

The coronavirus disease 2019 (COVID-19) pandemic is one of the greatest challenges for all countries worldwide. The increasing number of deaths has prompted the global scientific community to investigate the associations between the social determinants of health and mortality from the disease^1^.

Different circumstances can impact the health-disease process of individuals; for instance, people with greater social vulnerability have an increased risk of becoming ill^1,2^. Social, economic, political, demographic, and geographic contexts are determinants of this vulnerability, indicating how a community may respond to increased risk due to a pandemic^3^.

More severe outcomes, such as high mortality and case-fatality rates, are expected in countries with lower socioeconomic status^4^. Therefore, an understanding of the factors associated with the COVID-19 pandemic is essential to identify the dynamics and the effects of the disease on population groups to develop protective measures as part of public policies to mitigate the disease effects and social impacts^4^.

In this context, Brazil gained prominence during the pandemic due to a combination of factors: the disease magnitude, including high incidence, mortality, and case-fatality rates; failures in leadership and management of the pandemic by government agencies; failure to implement a mass testing programme, contact tracing, early quarantine, and isolation of positive cases; and low adherence to preventive interventions due to social and ethnic complexities^5^.

Long after the beginning of the pandemic and the development of vaccines, Brazil ranked first in the number of daily COVID-19-related deaths worldwide, reaching record numbers of notifications of new cases in a single day (115,228 cases) on 23 June 2021, and new deaths (4249 deaths) on 8 April 2021^6^. The numbers of deaths and new cases decreased and stabilised by 8% and 5%, respectively, in epidemiological weeks (EWs) 30 (25–31 July 2021) and 31 (1–7 August 2021), with moving averages of 989 deaths in EW 30 and 912 in EW 31^2^.

COVID-19-related mortality in Brazil is associated with significant disparities in access to healthcare services, as well as population age structure, comorbidities, and sex, with higher mortality in the older population; men, individuals with pre-existing health problems and between *pardos* and blacks^7^.

As the Brazilian population is quite diverse, the Brazilian Institute of Geography and Statistics (IBGE) classifies the population based on self-declaration into five categories: white, black, yellow, indigenous or *pardos*, the latter being a mixture of ethnicities^5^.

The burden on healthcare services, interruption of chronic diseases treatments, and patient resistance to seeking care due to fear of acquiring COVID-19 may also be correlated with the number of deaths^8^. The World Health Organization recommends the epidemiological surveillance of these factors to evaluate the direct and indirect effects of the pandemic^8^.

Therefore, this study analysed the spatio-temporal distributions of COVID-19 incidence, mortality, and case-fatality ratios, and their associations with social determinants of health in Brazil between March 2020 and September 2021.

## Results

**Figure 1:**
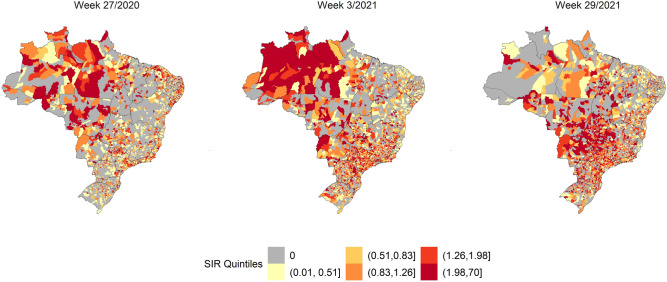
SIR Quintiles by Brazilian municipalities in weeks 27/2020, 3/2021, and 25/2021. The map was generated using public domain shapefiles provided by the Brazilian Institute of Geography and Statistics (https://geoftp.ibge.gov.br/organizacao_do_territorio/malhas_territoriais/malhas_municipais/municipio_2021/Brasil/BR/BR_Municipios_2021.zip). The figure was generated by the authors using the R 4.0.5 software^9^ and the packages ggplot2^10^, sf^11^ and tidyverse^12^.

Table 1 presents the numbers of patients with of SARS due to COVID-19 by sex, age group, and progression during the 2-year study period. More cases of SARS occurred in 2021. The disease was more frequent in men throughout the study period (55·6%). The highest proportion of SARS was reported in the 50–59-year age group (20·2%). The reported case-fatality rate was 33·4% and was higher in 2021 (33·7%) (Fig. 1).

**Table 1:** Number of SARS cases, Brazil, 2020 and 2021.

	2020 (N = 700,742)	2021 (N = 1,185,556)	Total (N = 1,886,298)
	N (%)	N (%)	N (%)
Sex			
Female	312,029 (44·5)	524,501 (44·2)	836,530 (44·3)
Male	388,565 (55·5)	660,901 (55·7)	1,049,466 (55·6)
Ignored	148 (0·0)	154 (0·0)	302 (0·0)
Age group			
0–4	6247 (0·9)	7847 (0·7)	14,094 (0·7)
5–9	3834 (0·5)	3946 (0·3)	7780 (0·4)
10–14	2748 (0·4)	3110 (0·3)	5858 (0·3)
15–19	4477 (0·6)	5709 (0·5)	10,186 (0·5)
20–29	27,360 (3·9)	48,283 (4·1)	75,643 (4·0)
30–39	65,436 (9·3)	138,450 (11·7)	203,886 (10·8)
40–49	95,544 (13·6)	209,030 (17·6)	304,574 (16·1)
50–59	123,886 (17·7)	256,205 (21·6)	380,091 (20·2)
60–69	143,080 (20·4)	226,430 (19·1)	369,510 (19·6)
70–79	124,450 (17·8)	168,519 (14·2)	292,969 (15·5)
80 and more	103,680 (14·8)	118,027 (10·0)	221,707 (11·8)
Evolution			
Healing	427,513 (64·6)	672,358 (63·1)	109,9871 (63·7)
Death	217,375 (32·9)	359,382 (33·7)	576,757 (33·4)
Death from other causes	3271 (0·5)	3683 (0·3)	6954 (0·4)
Ignored	13,438 (2·0)	30,667 (2·9)	44,105 (2·6)

**Figure 2:**
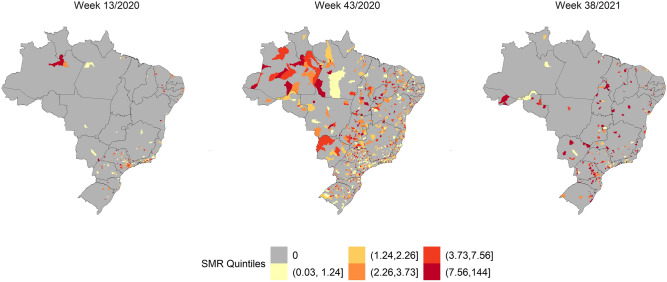
SMR quintiles by Brazilian municipalities in weeks 13/2020, 43/2021, and 38/2021. The map was generated using public domain shapefiles provided by the Brazilian Institute of Geography and Statistics (https://geoftp.ibge.gov.br/organizacao_do_territorio/malhas_territoriais/malhas_municipais/municipio_2021/Brasil/BR/BR_Municipios_2021.zip). The figure was generated by the authors using the R 4.0.5 software^9^ and the packages ggplot2^10^, sf^11^ and tidyverse^12^.

**Figure 3:**
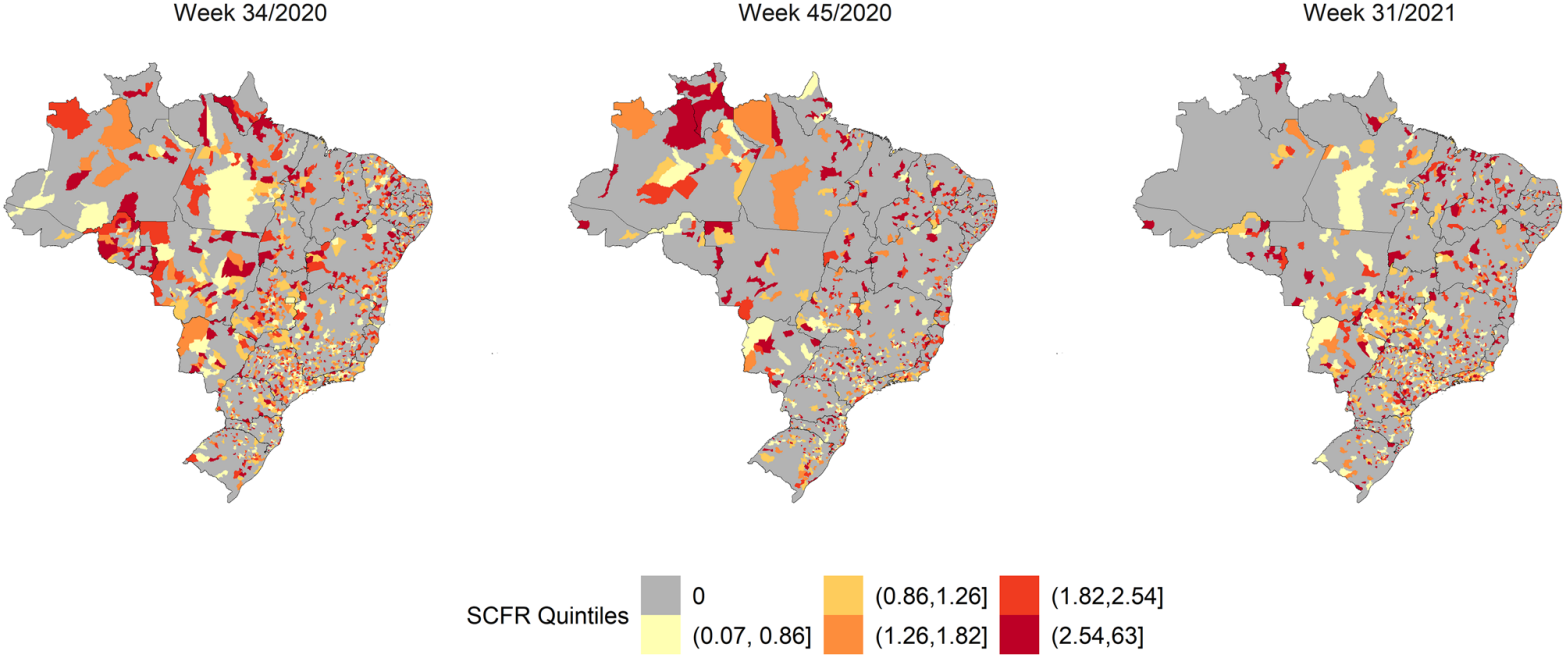
SCFR Quintiles by Brazilian municipalities in weeks 41/2020, 6/2021, and 24/2021. The map was generated using public domain shapefiles provided by the Brazilian Institute of Geography and Statistics (https://geoftp.ibge.gov.br/organizacao_do_territorio/malhas_territoriais/malhas_municipais/municipio_2021/Brasil/BR/BR_Municipios_2021.zip). The figure was generated by the authors using the R 4.0.5 software^9^ and the packages ggplot2^10^, sf^11^ and tidyverse^12^.

Figures 1, 2 and 3 show the SIR, SMR, and SCFR maps of the municipalities during three some selected weeks with high incidence, deaths, and case fatalities in Brazil. Figure S1 shows the distributions of SIR, SMR, and SCFR in all EW of the study period.

EW 27/2020, 3/2021, and 29/2021 showed the highest SIR of SARS in the country (Fig. 1). In the first week evaluated, the highest values were observed in the north region, in addition to the main Brazilian capitals. The disease then spread to the entire Brazilian countryside. By week 29/2021, the highest SIR was reported in the centre-west and south regions.

Figure 2 shows the highest SARS SMR in Brazil during EWs 13/2020, 43/2020, and 38/2021. Mortality increased throughout the territory over time (week 43 of October 2020), but not at the same intensity as that of the incidence. In the last weeks of the study, the SMR decreased and concentrated in discrete locations of all regions of the country.

Figure 3 shows the highest SARS SCFR in the country in EWs 34/2020, 45/2020, and 31/2021. The SCFR was high throughout the Brazilian territory in these three EWs.

The distributions of SIR, SMR, and SCFR over the study period are shown in Figs. S2, S3, and S4, respectively (supplementary material).

**Figure 4:**
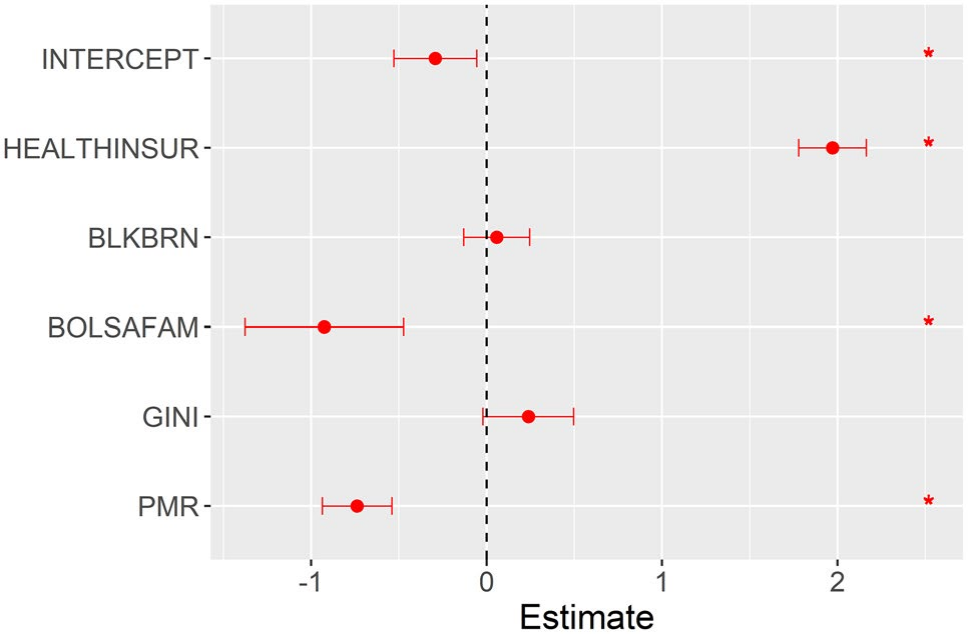
Results of the ZINB spatial–temporal regression model for the standardised incidence ratio of severe acute respiratory syndrome coronavirus 2.

**Figure 5:**
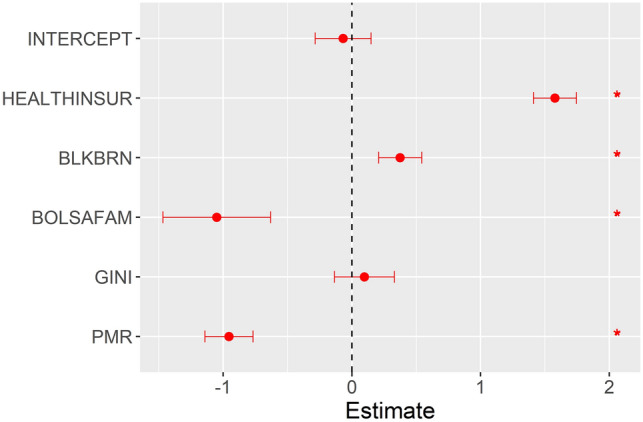
Results of the spatial–temporal ZINB regression models for the standardised mortality ratio of severe acute respiratory syndrome coronavirus 2.

**Figure 6:**
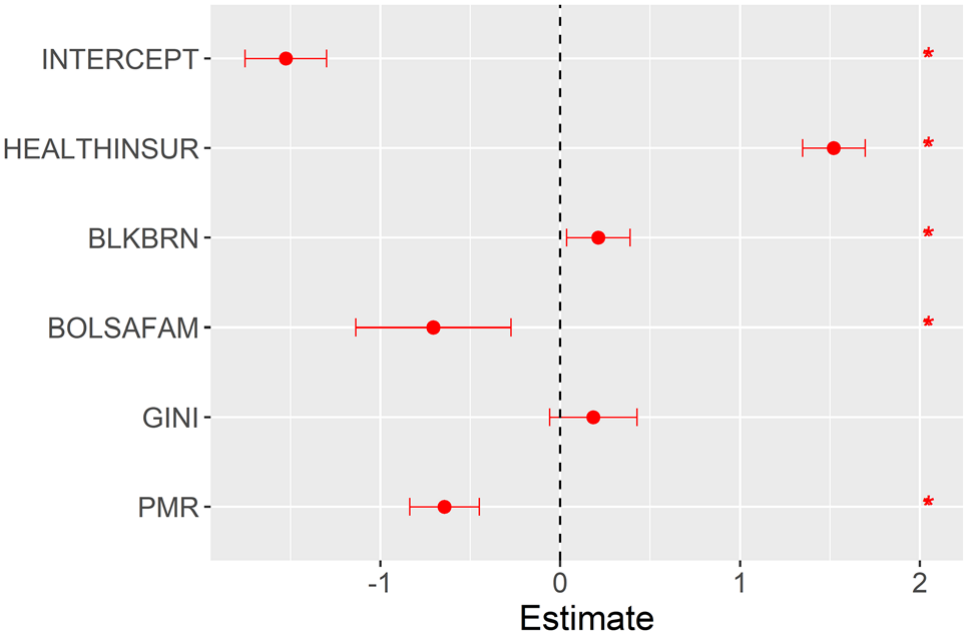
Results of the ZINB spatial–temporal regression models for the standardised case fatality ratio of severe acute respiratory syndrome coronavirus 2.

After producing a Spearman’s correlation matrix between the three outcomes analysed (SIR, SMR, and SCFR) and the study covariates, the covariates with significant correlations were selected (Table S3) and included in the model for each study outcome. Figures 4, 5 and 6 show the medians and 0.025 and 0.975 quantiles of the coefficients obtained with the INLA method used in the ZINB spatial–temporal regression model.

In the model with the SIR outcome (Fig. 4), the per capita Bolsa Família Programme benefits distributed in the municipality (BOLSAFAM) and the proportional mortality ratio (PMR) were inversely associated with the outcome, while the proportion of population covered by health insurance (HEALTHINSUR) and Gini index (GINI) were directly associated with the SIR outcome.

In the model with the SMR outcome (Fig. 5), BOLSAFAM and PMR were inversely associated with the outcome, similar to the findings for the model with the SIR outcome. HEALTHINSUR and proportion of *pardo* and Black population in the municipality (BLKBRN) were directly associated with the SMR outcome.

The model with the SCFR outcome (Fig. 6) showed similar results to those of the model with the SMR outcome. BOLSAFAM and PMR were inversely associated while HEALTHINSUR and BLKBRN were directly associated with the SCFR outcome.

**Figure 7:**
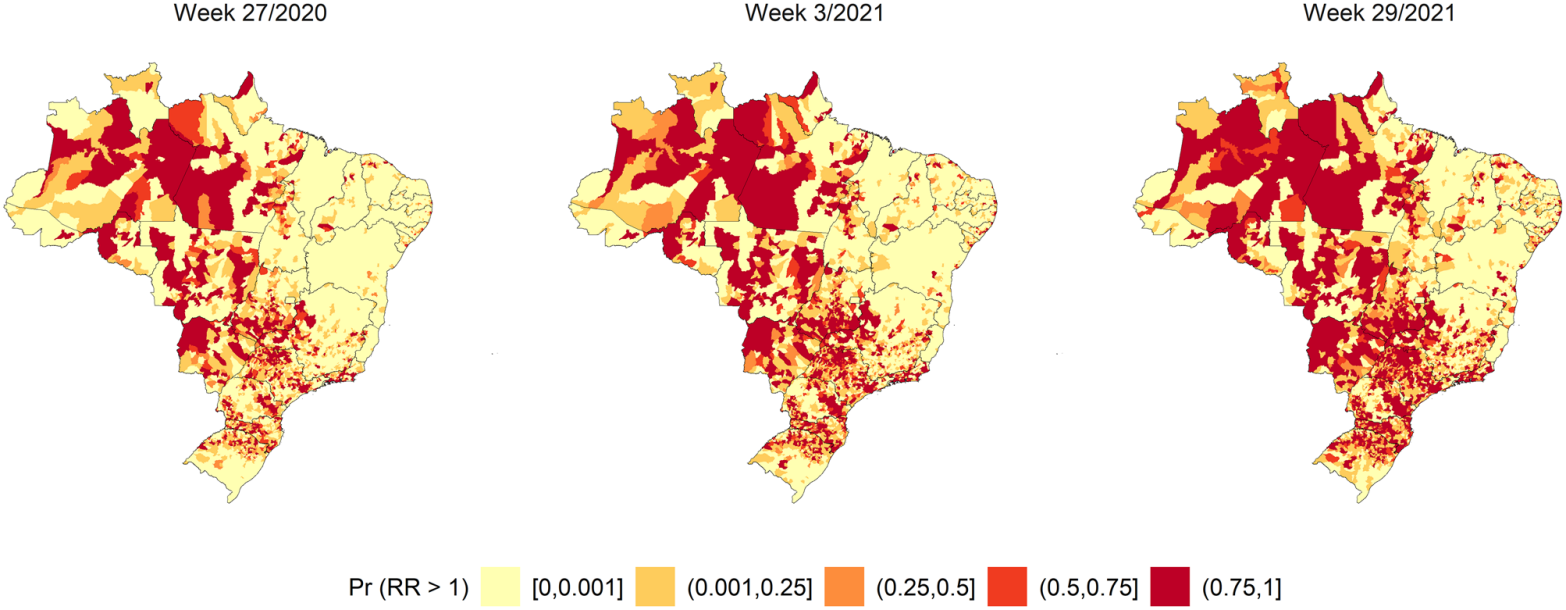
Relative risk of the ZINB spatial–temporal regression models for the standardised incidence ratio of severe acute respiratory syndrome coronavirus 2 in weeks 15/2020, 30/2021, 45/2020, 7/2021, 22/2021, and 38/2021. The map was generated using public domain shapefiles provided by the Brazilian Institute of Geography and Statistics (https://geoftp.ibge.gov.br/organizacao_do_territorio/malhas_territoriais/malhas_municipais/municipio_2021/Brasil/BR/BR_Municipios_2021.zip). The figure was generated by the authors using the R 4.0.5 software^9^ and the packages ggplot2^10^, sf^11^ and tidyverse^12^.

**Figure 8:**
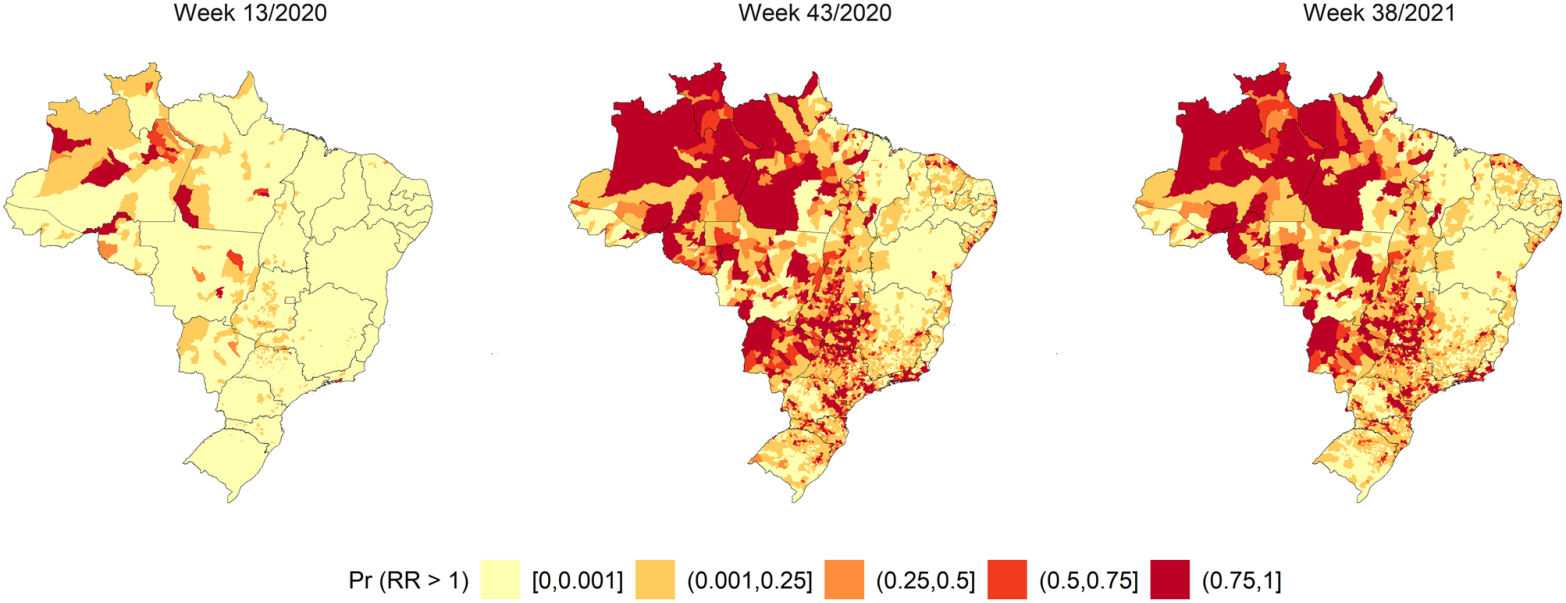
Relative risk of the ZINB spatial–temporal regression models for the standardised mortality ratio of severe acute respiratory syndrome coronavirus 2 in weeks 15/2020, 30/2021, 45/2020, 7/2021, 22/2021, and 38/2021. The map was generated using public domain shapefiles provided by the Brazilian Institute of Geography and Statistics (https://geoftp.ibge.gov.br/organizacao_do_territorio/malhas_territoriais/malhas_municipais/municipio_2021/Brasil/BR/BR_Municipios_2021.zip). The figure was generated by the authors using the R 4.0.5 software^9^ and the packages ggplot2^10^, sf^11^ and tidyverse^12^.

**Figure 9:**
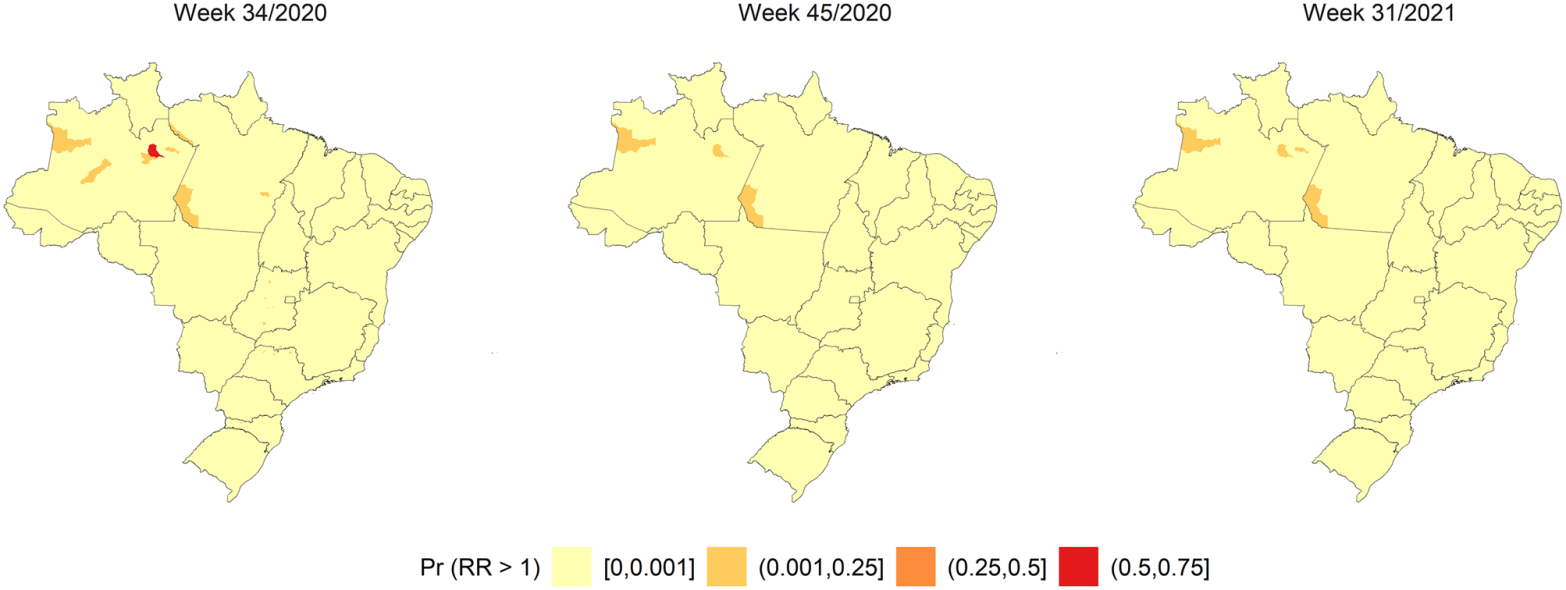
Relative risk of the ZINB spatial–temporal regression models for the standardised case fatality ratio of severe acute respiratory syndrome coronavirus 2 in weeks 15/2020, 30/2021, 45/2020, 7/2021, 22/2021, and 38/2021. The map was generated using public domain shapefiles provided by the Brazilian Institute of Geography and Statistics (https://geoftp.ibge.gov.br/organizacao_do_territorio/malhas_territoriais/malhas_municipais/municipio_2021/Brasil/BR/BR_Municipios_2021.zip). The figure was generated by the authors using the R 4.0.5 software^9^ and the packages ggplot2^10^, sf^11^ and tidyverse^12^.

The probabilities of excess risk are shown in Figs. 7, 8 and 9. The EWs were selected according to the SIR peaks.

Figure 7 shows that the highest proportion of excess risk for the SIR outcome started in the north region and part of the centre-west regions, later extending to the entire centre-west, southeast, and south regions. However, this excess risk was detected predominantly in the north region in all periods.

Figure 8 shows that the highest proportion of excess risk for the SMR outcome started in the north region and expanded to the centre-west, southeast, and south regions. However, the risk was detected predominantly in the north region in all periods.

Figure 9 shows that the highest proportion of excess risk for the SCFR outcome was obeserved in some municipalities in the north region and other regions of Brazil.

The RR results for the INLA models of SIR, SMR, and SCFR over the entire study period are presented in Figures S5, S6, and S7, respectively (supplementary material).

## Discussion

SIR, SMR, and SCFR were inversely associated with both the per capita Bolsa Família Programme benefits distributed in the municipality and the PMR. Several authors have demonstrated the positive impact of Bolsa Família, a cash transfer programme for vulnerable sectors of the Brazilian population, on the decreasing child mortality^13^, the incidence of leprosy in families living in municipalities with a high burden of the disease^14^, and maternal mortality^15^, and increasing the number of people recovering from tuberculosis^16^.

In this context, cash transfer programmes for socially vulnerable individuals, such as the Bolsa Família Programme, play a key role in protecting the most basic needs of people, including nutritional security^17^. In this study, both incidence and mortality were inversely associated with the per capita Bolsa Família Programme benefits, a finding that demonstrated the relevance of this welfare programme, especially in a pandemic.

PMR is a proxy for social development, in which the higher the PMR, the better the social development. One study showed an inverse association between PMR and the incidence of COVID-19^18^.

The three outcomes assessed in this study were directly associated with the proportion of population covered by health insurance. The associations of these outcomes with incidence, mortality, and case-fatality suggested that the access to private health services contributed to increased diagnosis and, consequently, greater case notification. However, the higher access to private health services was not enough to reduce deaths in municipalities with greater coverage of these services.

GINI, a measure of social inequality, was directly associated with the incidence of SARS due to COVID-19. Martines et al.^19^ reported a direct association between GINI and the RR of COVID-19 in Brazilian municipalities. Ribeiro et al.^20^ showed higher mortality from COVID-19 in areas in the city of São Paulo, SP, Brazil, with greater social inequalities. Islam et al.^21^ observed a cluster of municipalities with high mortality from COVID-19, high prevalence of chronic diseases, and high social vulnerability, especially in the southern USA. Furthermore, the COVID-19 pandemic tends to increase existing social inequalities. Mans and Mansmann^22^ used inequality indices to monitor the geographic differences in incidence, mortality, and case-fatality rates over the first 12 months of the COVID-19 pandemic worldwide, reporting an unfavourable GINI progression in all four continents since February 2020.

A previous study showed increasing COVID-19 incidence and mortality rates in all Brazilian federal units, with higher rates in those with greater economic inequality. The association between the GINI and the incidence of COVID-19 persisted even when the demographic and spatial aspects were considered^23^. Other studies on COVID-19 incidence and mortality supported the role of inequality as an important social determinant of health^19,24^.

The proportion of the Black and *pardo* population was directly associated with mortality and case-fatality ratios. The explanations for the racial differences in the COVID-19 mortality and case-fatality ratios tend to consider the multiple potentially associated factors that increase the risk of the disease, such as the type of occupation and prevalence of chronic conditions^7^. A study in some regions of Brazil showed that patients of White ethnicity had greater chances of surviving and being admitted to an intensive care unit to those of *pardo* ethnicity^5^. In the United States, the rates of COVID-19 diagnosis and death in counties with a high proportion of Black people were significantly higher than those in other counties, even after adjusting for confounding factors^25^. Some authors^3^ reported that the spatial clusters of social vulnerability were significantly associated with increased mortality rates due to COVID-19 and that a higher proportion of African Americans in Chicago had high levels of social vulnerability and several risk factors. At the individual level, Black^26^ and non-White people have a higher risk of mortality^27^.

Baptista et al. pointed out the high risk of mortality from COVID-19 in the elderly, especially men, with a reduction in the difference between genders after the second wave of the disease in Brazil^28^. Lima et al. delved into the topic, noting that even with the younger age profile, the North region and part of the northeastern coast, in general, had a higher risk of mortality and incidence of COVID-19, possibly due to the unfavorable health conditions in the region^29^. In the present study, the control for the effect of age on the calculation of incidence and mortality was carried out by standardizing cases and deaths by age group. However, even so, the North region stood out in terms of incidence and mortality measures, highlighting the relevance of investigating other effects that influence epidemiological measures.

Regarding the spatio-temporal progression of the three outcomes, the north region consistently showed a higher excess risk for COVID-19 incidence and mortality during the entire study period. The state of Amazonas, located in the north region of Brazil, had several problems during the pandemic. During the first wave of the disease (May 2020), Amazonas had an exponential increased number of deaths, leading to the collapse of the health system. Nevertheless, Manaus, the state’s capital and largest city, had begun to relax its social distancing requirements^30^. A study in October 2020 estimated that Manaus had an attack rate of 76%. However, heterogeneity in immune protection and population structure, in addition to poverty, poor public transportation, and challenges in the adoption of non-pharmaceutical measures, impaired the achievement of herd immunity. The Manaus tragedy shows that inadequate control of the SARS-CoV-2 spread can cause infection in a large part of the population, leading to high mortality^31^.

The impact of the COVID-19 pandemic reached several countries around the world, with high estimated declines in life expectancy at birth from the American continent to Europe^32^. In Brazil, the calculated reduction was 1.3 years in 2020, with the states of greater prominence in the northern region of the country, with a drop of up to 3.46 years in the state of Amazonas^33^.

The progression of the COVID-19 pandemic in Brazil was a tragedy that could have been avoided by establishing government policies, widespread testing and isolation of positive cases, mass vaccination, and observing non-pharmacological measures. This tragedy would have been worse if the Unified Health System, a public, free and accessible health system, had not prevented some deaths.

The main limitations of our study are related to the quality of the data and the gap in the socioeconomic information. Due to the use of secondary data sources, underreporting of cases and deaths from Covid-19 throughout the national territory can interfere with the incidence and mortality rates for the disease, and thus generate information that is not representative of the distribution of the disease in the population. In addition, some municipalities may have undergone changes in their socioeconomic characteristics, which could influence the results at the local level. However, the study assumed the non-occurrence of significant changes in the municipalities’ socioeconomic profile over the course of 12 years.

## Methods

### Design

Ecological studies use a population group as a unit of analysis, commonly delimited by a geographic area, to assess the possible relationship between socio-environmental and health characteristics^34^.

This analytical ecological study evaluated, at municipal level, the spatial and temporal association between demographic, socioeconomic, and healthcare variables and the three outcomes: incidence, mortality, and case-fatality ratios due to severe acute respiratory syndrome (SARS) caused by severe acute respiratory syndrome coronavirus 2 (SARS-CoV-2).

### Data collection

Information on cases and deaths associated with SARS due to COVID-19 in Brazil were obtained from the Influenza Epidemiological Surveillance Information System database and used to the calculate the incidence, mortality, and case-fatality ratios for severe cases.

The geographic units of analysis were the 5570 Brazilian municipalities. The temporal cut-off points were the EWs 13–53 of 2020 and 1–38 in 2021, corresponding to 22 March 2020 to 25 September 2021^35^. For analysis, the sequence of EWs started in 2020 and continued into 2021.

**Figure 10:**
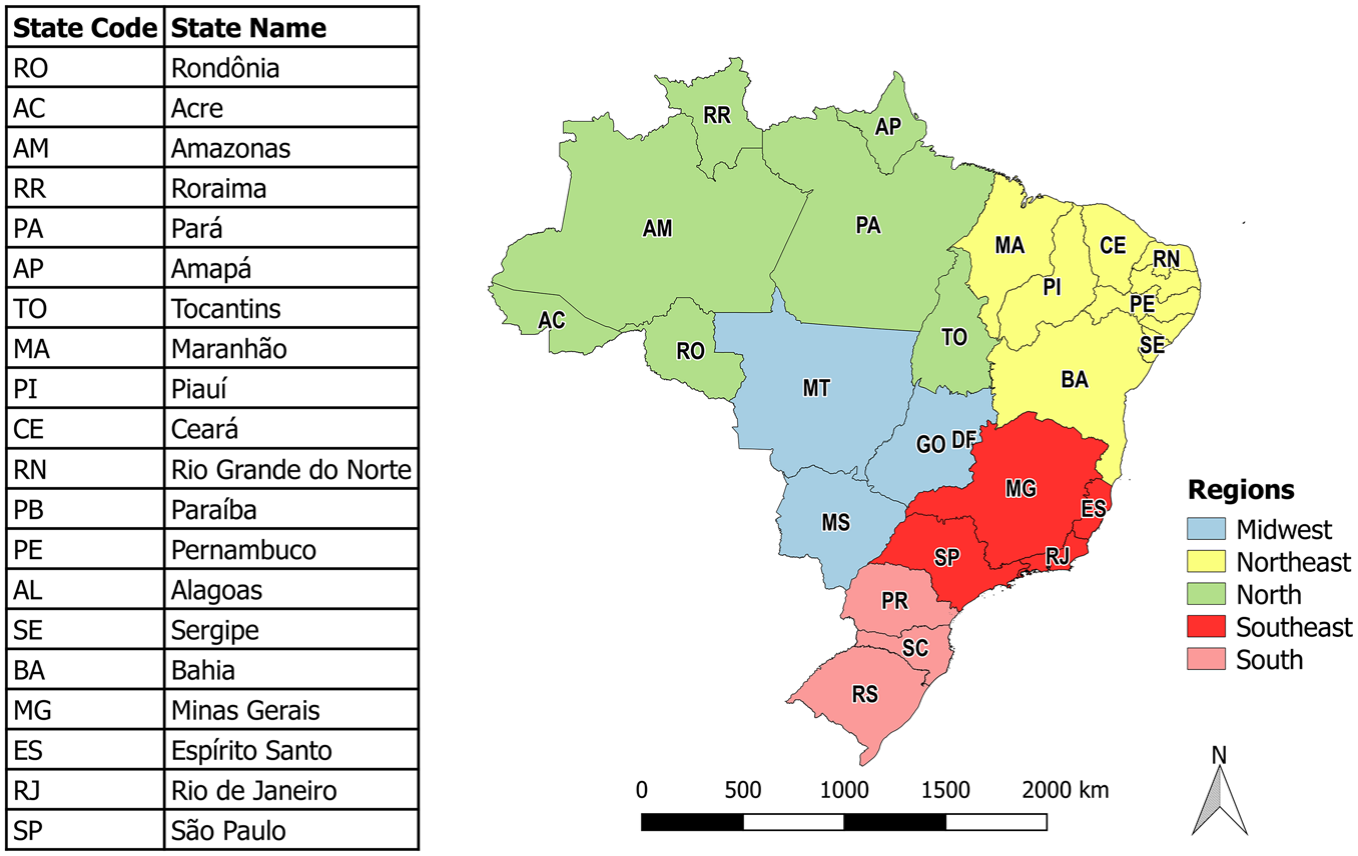
Administrative division of Brazil. The map was generated using public domain shapefiles provided by the Brazilian Institute of Geography and Statistics (https://geoftp.ibge.gov.br/organizacao_do_territorio/malhas_territoriais/malhas_municipais/municipio_2021/Brasil/BR/BR_Municipios_2021.zip). The figure was generated by the authors using the QGIS software, version 3.16.11 (https://qgis.org).

Figure 10 shows the administrative division of Brazil into 26 federative units (FUs) and the Federal District (DF). These FUs are administratively grouped into five regions.

To identify all cases of SARS due to COVID-19 in the database, we re-evaluated SARS cases with unknown aetiology. Among these cases, COVID-19 was considered in patients who tested positive for SARS-CoV-2 by reverse-transcriptase polymerase chain reaction (RT-PCR), who showed IgG, IgM, or IgA antibodies against this virus, and those with imaging test (chest computed tomography scan) findings typical of COVID-19, including the ground-glass opacity or reverse halo sign criteria adopted by the Brazilian Ministry of Health^36^.

### Data analysis

The heterogeneity of the distributions of outcomes in the Brazilian territory may produce biased risk estimates. In some municipalities, the number of cases was extremely low during the study period. Furthermore, the age structure of the population can also influence the results. Thus, the indirect standardization method was used to calculate the incidence, mortality, and fatality ratios of COVID-19^37^. Thus, we assessed whether the observed and expected values between the three outcomes for each municipality $(i)$ and EW $(t)$ differed from those of the reference population. The reference population in this study had the same age distribution as that of Brazilian individuals in 2020. The age groups were 0–4, 5–9, 10–14, 15–19, 20–29, 30–39, 40–49, 50–59, 60–69, 70–79, and 80 years or older. The expected number of cases and deaths, as well as the case-fatality rate per age group in each municipality for each EW, was calculated by multiplying the population values of each municipality by the specific incidence, mortality, or case-fatality rates. For $i \left( {i = 1, \ldots , 5570} \right)$ municipalities, $t \left( {t = 13, \ldots , 64} \right)$ weeks, where y_c_, y_d_ are the number of cases and deaths in municipality i and week t; E_c_ (1), E_d_ (2), and E_l_ (3) are the expected number of cases, deaths, and SARS case fatalities in municipality i and week j; and P_itn_ is the reference population, as follows^38^.

1 $$E_{{c_{{it}} }} = P_{{it_{n} }} \left( {\frac{{\mathop \sum \nolimits_{{i = 1}}^{n} \mathop \sum \nolimits_{{t = 1}}^{T} y_{{cit_{{1, \ldots ,n}} }} }}{{\mathop \sum \nolimits_{{i = 1}}^{n} \mathop \sum \nolimits_{{t = 1}}^{T} p_{{it_{{1, \ldots ,n}} }} }}} \right)$$

2 $$E_{{d_{{it}} }} = P_{{it_{n} }} \left( {\frac{{\mathop \sum \nolimits_{{i = 1}}^{n} \mathop \sum \nolimits_{{t = 1}}^{T} y_{{dit_{{1, \ldots ,n}} }} }}{{\mathop \sum \nolimits_{{i = 1}}^{n} \mathop \sum \nolimits_{{t = 1}}^{T} p_{{it_{{1, \ldots ,n}} }} }}} \right)$$

3 $$E_{{l_{{it}} }} = P_{{it_{n} }} \left( {\frac{{\mathop \sum \nolimits_{{i = 1}}^{n} \mathop \sum \nolimits_{{t = 1}}^{T} y_{{dit_{{1, \ldots ,n}} }} }}{{\mathop \sum \nolimits_{{i = 1}}^{n} \mathop \sum \nolimits_{{t = 1}}^{T} y_{{cit_{{1, \ldots ,n}} }} }}} \right)$$

After calculating the expected case, death, and case-fatality values, the relative risk estimators, standardised incidence rate (SIR), standardised mortality rate (SMR), and standardised case-fatality ratio (SCFR) were determined.

A Bayesian space–time model was adjusted using integrated nested Laplace approximation (INLA) to evaluate the risk of SARS by municipality and EW^39^. The outcomes of each municipality *i* to EW *t* followed a Poisson distribution. If the model showed overdispersion, a negative binomial distribution was used. Since the pandemic spread at different moments in time among the municipalities, zero-inflated (zero-inflated Poisson [ZIP] and zero-inflated negative binomial [ZINB]) models were used. The selection of the probability distribution that best fitted the data was based on the smallest Watanabe-Akaike information criterion (WAIC) by comparing the models to the intercept^40^ (the statistical results are showed in Table S1). The Besag-York-Mollié model (BYM2), a variation of the conditional autoregressive (CAR) model, was included to characterise the spatial dependence, in which events occurring in neighbouring areas had greater correlations than those occurring in distant areas^41,42^. A second-order random walk model (RW2) was used to calculate the time dependence, which was characterised by consecutive time units presenting similar risk estimates (the results of the comparison between models are included in Table S2). The space–time interaction was included with an unstructured random effect term^43^. Thus, the INLA model with the space–time structure was constructed using the number of cases and deaths as independent variables and the expected value as an offset in a logarithmic scale.

For assessing the degree of uncertainty of RR, we calculate the probability of excess risk > 1. The probabilities of excess risk greater than or equal to 75% indicate an RR high certainty to detect true raised-risk municipality^40^.

Demographic, socioeconomic, and healthcare covariates were included to investigate their possible associations with the outcomes. These covariates originated from different databases, which are detailed in Table 2.

**Table 2:** Study covariates, data source, measures, and year of reference.

Description of variables	Acronym	Data source	Reference
Total estimated population of the municipality	ESTIMPOP	DATASUS—Resident population—estimates for TCU	2019
Proportion of the *pardo* and Black population in the municipality	BLKBRN	DATASUS—Resident population—estimates for TCU	2019
Average monthly number of registered physicians by municipality	DOCTRS	Ministry of Health, Brazilian National Register of Health Institutions, CNES	January-June 2020
Average monthly number of registered nurses per municipality	NURS	Ministry of Health, Brazilian National Register of Health Institutions, CNES	January-June 2020
Proportion of population covered by health insurance	HEALTHINSUR	National Supplementary Health Agency	March 2020
Per capita amount of Bolsa Família Programme benefits distributed in the municipality	BOLSAFAM	Ministry of Citizenship, Secretariat	2020–2021
Gini index	GINI	Brazilian Institute of Geography and Statistics—Demographic Census	2010
PMR (Swaroop and Uemura index)	PMR	Mortality Information System	2018
Human development index (HDI)	HDI	Atlas of Human Development	2010
Proportion of the population aged ≥ 15 years with 0–4 years of schooling	0–4 YEARSSCHOL	Brazilian Institute of Geography and Statistics—Demographic Census	2010
Proportion of the population aged ≥ 15 years with 5–8 years of schooling	5–8 YEARSSCHOL	Brazilian Institute of Geography and Statistics—Demographic Census	2010
Proportion of population aged ≥ 15 years with nine or more years of schooling	9 + YEARSSCHOL	Brazilian Institute of Geography and Statistics—Demographic Census	2010

Due to the Covid-19 pandemic, from April to December 2020, beneficiary families eligible for the Bolsa Família Programme received emergency assistance, a supplement to the benefit provided by the federal government.

Due to the large number of covariates, they were initially selected following the epidemiological criteria and statistical correlations. Spearman’s correlation coefficients with a significance level of 5% were used to evaluate the correlations between the outcomes and covariates (the Spearman’s correlation matrix is included in Table S3).

Furthermore, the collinearity among the selected covariates was tested using the variation inflation factor of the linear model. Collinearity was considered present for tolerance values < 10^44^. All analyses were performed using R statistical software version 4.0.5.

## Supplementary Information

Supplementary Information 1.

Supplementary Information 2.

Supplementary Information 3.

Supplementary Information 4.

Supplementary Information 5.

Supplementary Information 6.

Supplementary Information 7.

Supplementary Information 8.

## Data Availability

The source code and data necessary for the replication of our results and numbers will be available upon request to the corresponding author by email caducer@gmail.com. All data come from public sources and consist of aggregates (hence no individual data are included).
