# Supplementary figures and images for: Spatial–temporal distribution of incidence, mortality, and case-fatality ratios of coronavirus disease 2019 and its social determinants in Brazilian municipalities
Source: Sci Rep. 2023 Mar 13;13:4139. doi: 10.1038/s41598-023-31046-4 (PMC10009864; doi:10.1038/s41598-023-31046-4)

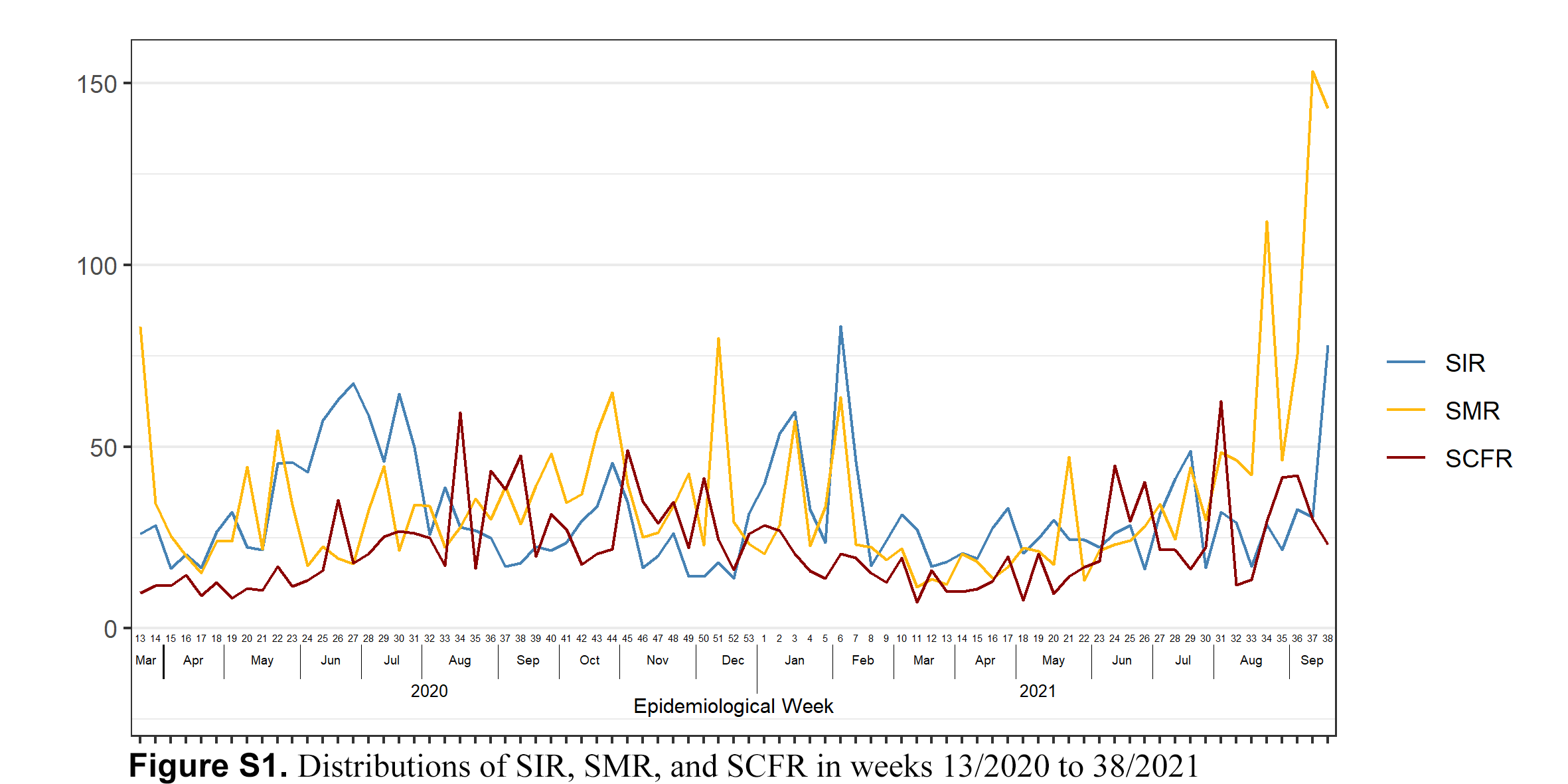

Supplement: Supplementary file 1 — Supplementary Information 1. [file 41598_2023_31046_MOESM1_ESM.tiff]

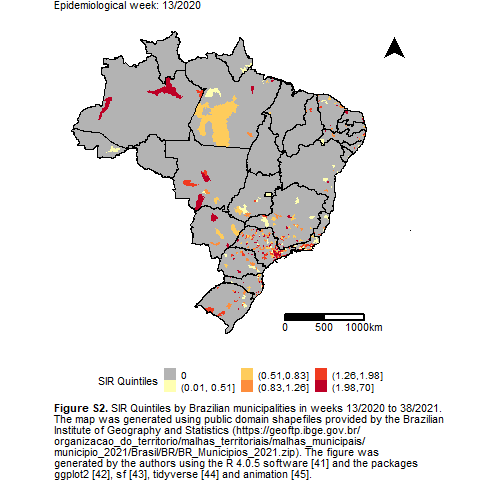

Supplement: Supplementary file 2 — Supplementary Information 2. [file 41598_2023_31046_MOESM2_ESM.gif]

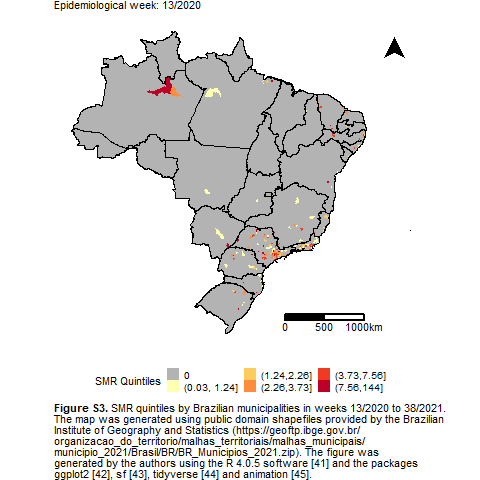

Supplement: Supplementary file 3 — Supplementary Information 3. [file 41598_2023_31046_MOESM3_ESM.gif]

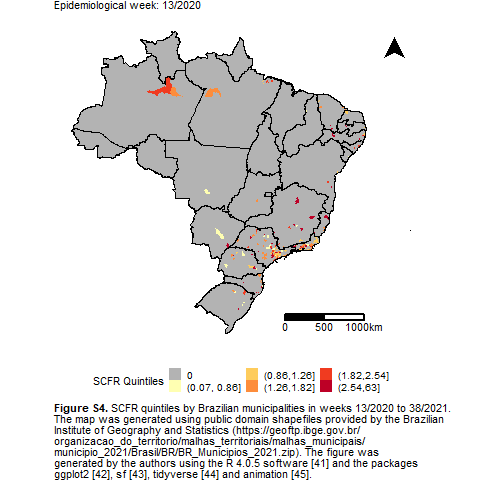

Supplement: Supplementary file 4 — Supplementary Information 4. [file 41598_2023_31046_MOESM4_ESM.gif]

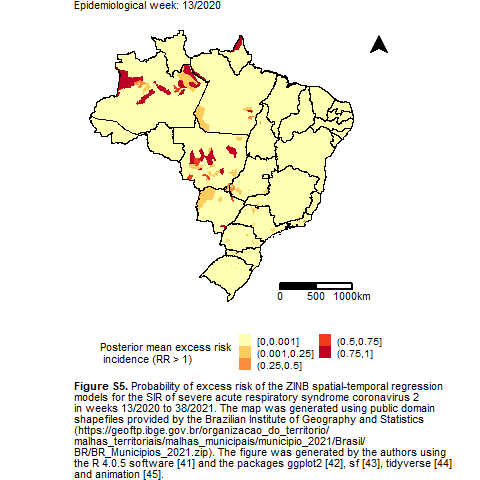

Supplement: Supplementary file 5 — Supplementary Information 5. [file 41598_2023_31046_MOESM5_ESM.gif]

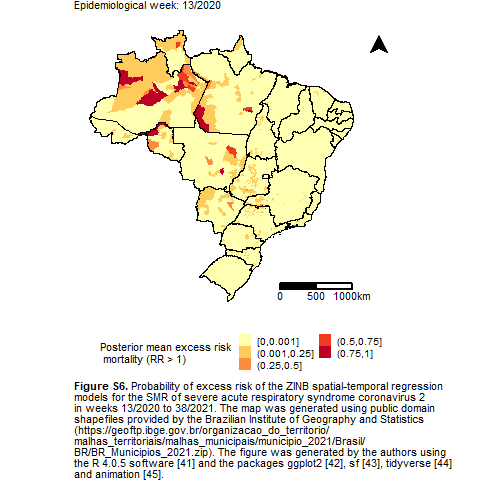

Supplement: Supplementary file 6 — Supplementary Information 6. [file 41598_2023_31046_MOESM6_ESM.gif]

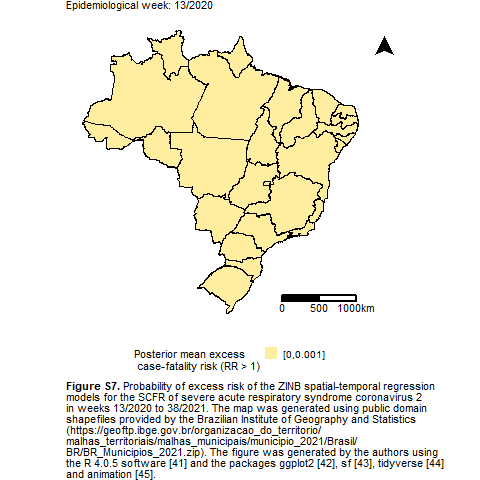

Supplement: Supplementary file 7 — Supplementary Information 7. [file 41598_2023_31046_MOESM7_ESM.gif]
