# Supplementary material for: Spatial–temporal distribution of incidence, mortality, and case-fatality ratios of coronavirus disease 2019 and its social determinants in Brazilian municipalities
Source: Sci Rep. 2023 Mar 13;13:4139. doi: 10.1038/s41598-023-31046-4 (PMC10009864; doi:10.1038/s41598-023-31046-4)
Supplement: Supplementary file 8 — Supplementary Information 8. [file 41598_2023_31046_MOESM8_ESM.docx]

**Supplementar Table S1 - WAIC and DIC models**

| Model | WAIC value | DIC value |
| --- | --- | --- |
| SIR | 1179317 | 1178536 |
| SMR | 692156 | 691854 |
| SCFR | 691845 | 691541 |

**Supplementar Table S2 - Contribution percentage of each random effect evaluated by the explained variance in the models**

|  | Models | | |
| --- | --- | --- | --- |
| Random effect | SIR (%) | SMR (%) | SCFR (%) |
| Municipality (IID) | 28,1 | 24,9 | 15,7 |
| Municipality (BYM2) | 66,2 | 72,5 | 77,3 |
| Week (IID) | 1,7 | 1,4 | 3,0 |
| Week (RW2)** | 4,0 | 1,2 | 4,0 |

IID: independent and identically distributed

RW2: structured temporal effect distributed as a random walk of first order

**Supplementar Table S3 – Spearman’s correlation matrix between the three outcomes analysed (SIR, SMR, and SCFR) and the study covariates**

|  | SMR | SCFR | BLKBRN | DOCTRS | NURS | HEALTHINSUR | BOLSAFAM | GINI | PMR | HDI | EDUC0TO4 | EDUC5TO8 | EDUC9 |
| --- | --- | --- | --- | --- | --- | --- | --- | --- | --- | --- | --- | --- | --- |
| SIR | 0.679* | 0.637* | -0.117* | 0.201* | 0.085* | 0.281* | -0.199* | -0.006* | -0.002 | 0.248* | -0.269* | -0.032* | 0.288* |
| SMR |  | 0.982* | -0.063* | 0.198* | 0.077* | 0.245* | -0.145* | 0.042* | -0.034* | 0.205* | -0.238* | -0.054* | 0.265* |
| SCFR |  |  | -0.051* | 0.194* | 0.071* | 0.236* | -0.131* | 0.053* | -0.041* | 0.192* | -0.228* | -0.056* | 0.258* |
| BLKBRN |  |  | -0.399* |  | 0.452* | 0.538* | -0.443* | -0.155* | 0.245* | 0.543* | -0.506* | 0.004* | 0.502* |
| DOCTRS |  |  | -0.135* |  |  | 0.212* | -0.145* | -0.023* | 0.140* | 0.265* | -0.219* | -0.075* | 0.258* |
| NURS |  |  |  |  |  | -0.591* | 0.765* | 0.514* | -0.583* | -0.730* | 0.579* | -0.167* | -0.469* |
| HEALTHINSUR |  |  |  |  |  |  | -0.741* | -0.381* | 0.350* | 0.832* | -0.765* | 0.079* | 0.757* |
| BOLSAFAM |  |  |  |  |  |  |  | 0.472* | -0.433* | -0.869* | 0.763* | -0.128* | -0.687* |
| GINI |  |  |  |  |  |  |  |  | -0.400* | -0.421* | 0.324* | -0.165* | -0.268* |
| PMR |  |  |  |  |  |  |  |  |  | 0.444* | -0.288* | 0.092* | 0.229* |
| HDI |  |  |  |  |  |  |  |  |  |  | -0.890* | 0.030* | 0.889* |
| EDUC0TO4 |  |  |  |  |  |  |  |  |  |  |  | -0.208* | -0.904* |
| EDUC5TO8 |  |  |  |  |  |  |  |  |  |  |  |  | -0.069* |

* Significant values at 5% level
